# Capillary pCO_2_ helps distinguishing idiopathic pulmonary arterial hypertension from pulmonary hypertension due to heart failure with preserved ejection fraction

**DOI:** 10.1186/s12931-015-0194-6

**Published:** 2015-03-10

**Authors:** Karen M Olsson, Lisa Sommer, Jan Fuge, Tobias Welte, Marius M Hoeper

**Affiliations:** Department of Respiratory Medicine and German Center of Lung Research (DZL), Hannover Medical School, 30623 Hannover, Germany

**Keywords:** Hypertension, Pulmonary, Left heart disease, Diastolic dysfunction, HFpEF, Hypocarbia, pCO_2_, Carbon dioxide

## Abstract

**Rationale:**

The demographics of patients with idiopathic pulmonary arterial hypertension (IPAH) are changing and this diagnosis is increasingly being made in older patients. However, diagnostic misclassifications are common as it may be difficult to differentiate between IPAH and pulmonary hypertension due to heart failure with preserved ejection fraction (PH-HFpEF). We investigated the hypothesis that the capillary pCO_2_ (p_c_CO_2_) may help distinguishing between idiopathic pulmonary arterial hypertension (IPAH) and pulmonary hypertension due to heart failure with preserved ejection fraction (PH-HFpEF).

**Methods:**

In a cross-sectional study, we retrospectively assessed p_c_CO_2_ levels (obtained from arterialized capillary blood at the time of diagnosis) from patients with IPAH or PH-HFpEF, respectively. Receiver operated characteristics (ROC) were used to determine the p_c_CO_2_ level providing the best discrimination between these two conditions. P_c_CO_2_ values were considered helpful if they were associated with a negative predictive value >0.9 to excluded either IPAH or PH-HFpEF.

**Results:**

The study enrolled 185 patients, 99 with IPAH (74% female; age 47 ± 17 years; body mass index 26 ± 5 kg/m^2^, PAPm 53 ± 12 mmHg, PAWP 8 ± 3 mmHg), and 86 with PH-HFpEF (64% female; age 69 ± 10 years; body mass index 30 ± 6 kg/m^2^, PAPm 47 ± 10 mmHg, PAWP 21 ± 5 mmHg). P_c_CO_2_ at time of diagnosis was 33 ± 4 mmHg in the IPAH group and 40 ± 5 mmHg in the PH-HFpEF group (p < 0.001), respectively. According to ROC analysis, a p_c_CO_2_ of 36 mmHg was the best discriminator between both entities with an area under curve of 0.87 (p < 0.001). The likelihood of PH-HFpEF was <10% in patients with a P_c_CO_2_ < 34 mmHg, whereas the likelihood of IPAH was <10% in patients with a P_c_CO_2_ > 41 mmHg.

**Conclusions:**

P_c_CO_2_ levels were significantly lower in IPAH compared to PH-HFpEF and may provide useful information in differentiating between both conditions.

## Introduction

According to the current classification, pulmonary hypertension (PH) is divided into 5 distinct groups: (i) pulmonary arterial hypertension (PAH), (ii) PH due to left heart disease, (iii) PH due to lung disease and/or hypoxia, (iv) chronic thromboembolic pulmonary hypertension (CTEPH), and (v) PH with unclear multifactorial mechanisms [1]. For most patients with PH, the diagnostic classification is straightforward but in occasional patients, the distinction between some of these conditions may be difficult.

An increasing diagnostic challenge in the work-up of patients with PH is the discrimination between idiopathic PAH (IPAH) and PH due to heart failure with preserved ejection fraction (PH-HFpEF). The current criteria for the distinction between IPAH and PH-HFpEF have limitations [2,3]. By definition, patients with IPAH have pre-capillary PH, i.e. a pulmonary artery wedge pressure (PAWP) or a left ventricular end-diastolic pressure (LVEDP) ≤15 mmHg, whereas patients with PH-HFpEF are characterized by post-capillary PH as defined by a PAWP/LVEDP >15 mmHg [2]. However, the invasive measurements of the left ventricular filling pressures can be misleading, both for technical as well as for physiological reasons [4]. Hence, PAWP/LVEDP measurements may yield values >15 mmHg in patients with PAH and - arguably more common - values ≤15 mmHg in patients with HFpEF, especially if left heart disease is optimally treated [5-7].

Thus, a single PAWP/LVEDP cut-off value is not always sufficient to allow an accurate diagnosis of pre- or post-capillary PH in each individual patient. This distinction, however, is of fundamental practical importance as the treatment of IPAH differs substantially from the treatment of patients with PH-HFpEF [8].

In the past, this problem was less evident as IPAH was originally considered predominantly a disease of younger women, and these patients are usually not at risk for developing HFpEF. More recently, however, IPAH is increasingly diagnosed in older patients, many of whom presenting with risk factors for developing left heart disease [9-11]. In a recently published report United Kingdom Pulmonary Hypertension registry, 13.5% of the patients were diagnosed with IPAH at an age >70 years, and in the European-based COMPERA registry, this proportion was even 50% [9,11]. It is possible that some of these patients were misclassified. Several conditions may mimic PAH and among those, HFpEF is the most common [2]. However all of the older patients in the abovementioned registries had a pulmonary arterial wedge pressure (PAWP) ≤15 mmHg, which – in a strict sense – would exclude a diagnosis of PH-HFpEF [9,11].

Hence measuring PAWP/LVEDP alone is not always sufficient to delineate IPAH from PH-HFpEF, and a comprehensive diagnostic assessment is required in order to ensure an accurate distinction between these two conditions. Risk factors for HFpEF include an older age, obesity, hypertension, diabetes and coronary heart disease [2,3]. The presence of echocardiographic signs of left ventricular diastolic dysfunction including an enlarged left atrium as well as the presence of permanent atrial fibrillation increase the likelihood of HFpEF, but none of these features excludes a diagnosis of IPAH.

It would be useful to have additional non-invasive variables that help distinguishing IPAH from PH-HFpEF. One potential candidate could be capillary pCO_2_ (p_c_CO_2_). P_c_CO_2_ can be obtained from arterialized earlobe sampling and accurately reflects arterial pCO_2_ [12-15]. Hyperventilation at rest and during exercise is a known feature of heart failure and PAH [16-19]. The mechanisms causing hyperventilation in these patients are incompletely understood, but increased physiologic dead space and, probably more importantly, increased chemosensitivity seem to play an important role [17,20]. Capillary p_c_CO_2_ tends to be more profoundly reduced in patients with IPAH [16,21] than in patients with PH-HFpEF [20,22]. Hence, we hypothesized that p_c_CO_2_ measurements may be helpful to discriminate between both conditions.

## Methods

Since April 2012, the Pulmonary Hypertension Clinic at Hannover Medical School has implemented an electronic database capturing all patients treated for PH. We used this database for a cross-sectional analysis of patients with well characterized IPAH or PH-HFpEF, respectively, based on the diagnostic criteria listed below. All variables analyzed and presented in this manuscript were obtained at the time of diagnosis, i.e. the time of the first diagnostic right heart catheterization.

General inclusion criteria for both groups were a body mass index <40 kg/m^2^, normal or near normal pulmonary function test results including a total lung capacity >80% predicted, a forced expiratory capacity in 1 s >60% predicted, a diffusion capacity for carbon monoxide >40% predicted, the absence or more than mild parenchymal abnormalities on chest computed tomography, and the need for non-invasive ventilation support for sleep-related breathing disorders. CTEPH was ruled out by ventilation-perfusion scintigraphy, and pulmonary angiography if needed, in all patients. All patients underwent right heart catheterization because of suspected severe PH or PAH, respectively, at the time of diagnosis with determination of right atrial pressure, mean pulmonary artery pressure (PAPm), PAWP and mixed venous oxygen saturation (SvO_2_). The pressure transducer was set at mid-thoracic level for all procedures. Cardiac output was measured by thermodilution; pulmonary vascular resistance (PVR), cardiac index (CI), transpulmonary gradient (TPG) and diastolic pulmonary gradient (DPG) were calculated by standard formula.

Patients with IPAH were included if they fulfilled the following criteria: PAPm ≥25 mmHg, PAWP ≤15 mmHg, PVR >240 dyn · s · cm^−5^, sinus rhythm at time of diagnosis, left ventricular ejection fraction >60% and normal size of the left atrium on echocardiography. A diagnosis of PH-HFpEF was based on the following criteria: PAPm ≥25 mmHg, PAWP >15 mmHg, left ventricular ejection fraction >50%, normal end-systolic and end-diastolic left ventricular diameters, and signs of diastolic dysfunction including the presence of an enlarged left atrium on echocardiography.

All patients provided written informed consent and the study was approved by the local ethics committee.

### Right heart catheterization

Right heart catheterizations were performed via a jugular approach following a standardized protocol. The pressure transducer was zeroed at the mid-thoracic level and all pressure readings were done at end-expiration [23]. Cardiac output was measured by thermodilution technique with the reported value being the average of at least three recordings with less than 10% variation.

### Blood gas analyses

Experienced technicians obtained arterialized capillary blood gases from earlobes after a resting period ≥10 min while patients were breathing room air. The blood samples were analyzed without delay using a standard device (Radiometer, Copenhagen).

### Statistical analysis

Data are shown as mean ± SD, unless indicated otherwise. For comparison of the two patient populations, Fisher’s exact test, Chi-square test and two-sided paired T-test were used as appropriate. Potential associations between p_c_CO_2_ and clinical variables were assessed with Pearson’s correlation analysis and two-sided testing for significance. In order to identify the p_c_CO_2_ level with the highest power to discriminate between IPAH and PH-HFpEF, receiver operated characteristics (ROC) curves were drawn and the area under the curve (AUC) was calculated. The cut-off value that resulted in the highest product of sensitivity and specificity was considered the best diagnostic p_c_CO_2_ value. Sensitivity, specificity, positive predictive values and negative predictive values were calculated assuming an equal pre-test probability of both conditions. P_c_CO_2_ values were arbitrarily considered useful for diagnostic purposes if they were associated with a negative predictive value >0.9 to excluded either IPAH or PH-HFpEF.

## Results

The study enrolled 185 patients; 99 with IPAH and 86 with PH-HFpEF. The patient characteristics are shown in Table 1. Compared to patients with PH-HFpEF, patients with IPAH were younger, had a lower body mass index and a lower likelihood of diabetes, while exercise capacity was less compromised. On right heart catheterization, patients with IPAH had higher values of PAPm and PVR whereas cardiac output, cardiac index, and right atrial pressures were lower. The arterial oxygen tension (paO_2_) was mildly reduced in both groups.

**Table 1:** **Patient characteristics at the time of diagnosis**

	**IPAH (n = 99)**	**PH-HFpEF (n = 86)**	**p-value**
Age (years)	47 ± 17	69 ± 10	<0.001
Female (%)	74	64	0.101
Body mass index (kg/m^2^)	26 ± 5	30 ± 6	<0.001
Diabetes (%)	19	57	<0.001
NYHA II/III/IV (n)	38/60/1	8/77/1	n/a
6 min walking distance (m)	386 ± 136	276 ± 117	<0.001
Right atrial pressure (mmHg)	7 ± 5	13 ± 5	<0.001
PAPm (mmHg)	53 ± 12	47 ± 10	<0.001
PAWP (mmHg)	8 ± 3	21 ± 5	<0.001
Transpulmonary gradient (mmHg)	46 ± 13	25 ± 10	<0.001
Diastolic gradient (mmHg)	24 ± 11	10 ± 8	<0.001
CO (L/min)	3.9 ± 1.2	4.9 ± 1.3	<0.001
CI (L/min/m^2^)	2.1 ± 0.6	2.5 ± 0.7	<0.001
PVR (dyn · s · cm^−5^)	1,017 ± 416	471 ± 218	<0.001
SvO_2_ (%)	64 ± 9	63 ± 8	0.368
P_c_aO_2_ (kPa)	9.5 ± 1.5	8.7 ± 1.2	0.039
P_c_aO_2_ (mmHg)	71 ± 11	65 ± 9	
P_c_aCO_2_ (kPa)	4.4 ± 0.5	5.3 ± 0.7	<0.001
P_c_aCO_2_ (mmHg)	33 ± 4	40 ± 5	

**Figure 1:**
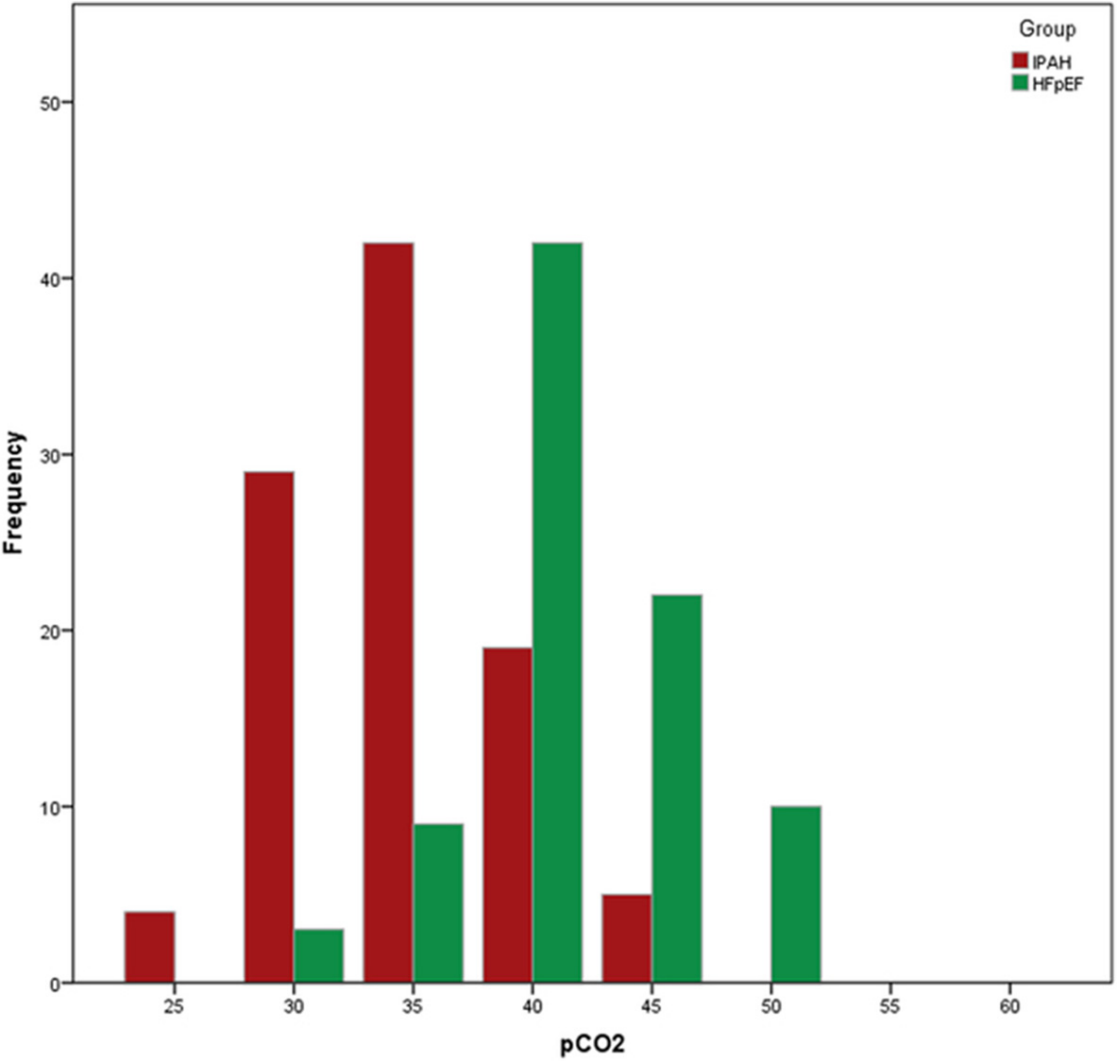
**Histogram showing the distribution of capillary pCO**_**2**_ **in patients with idiopathic pulmonary arterial hypertension (red) and patients with pulmonary hypertension due to heart failure with preserved ejection fraction (green) in 5 mmHg intervals.**

**Figure 2:**
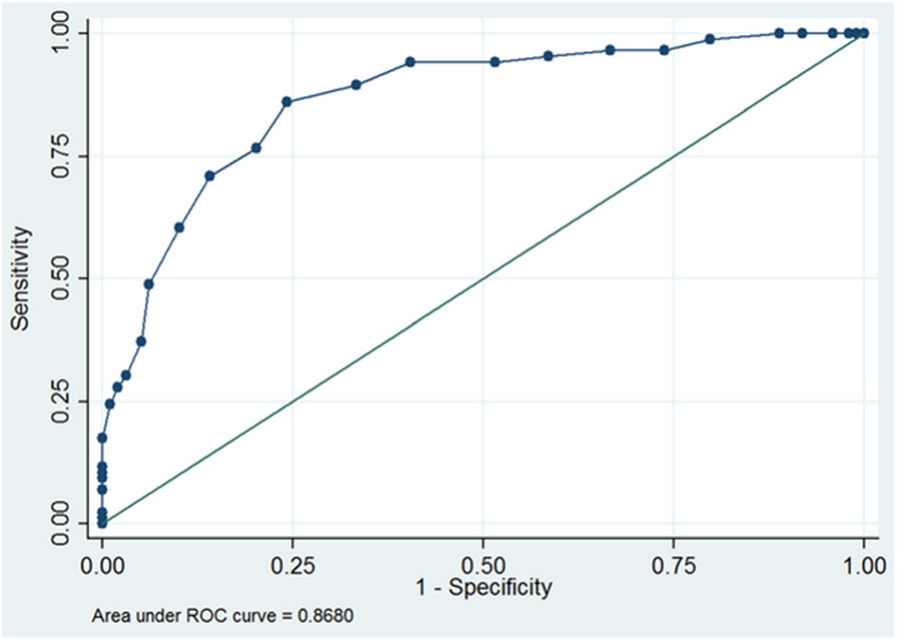
**Receiver operated characteristics (ROC) curve showing the diagnostic performance of p**_**c**_ **CO**_**2**_ **in distinguishing idiopathic pulmonary arterial hypertension (IPAH) from pulmonary hypertension in patients with heart failure and preserved ejection fraction (PH-HFpEF).**

**Figure 3:**
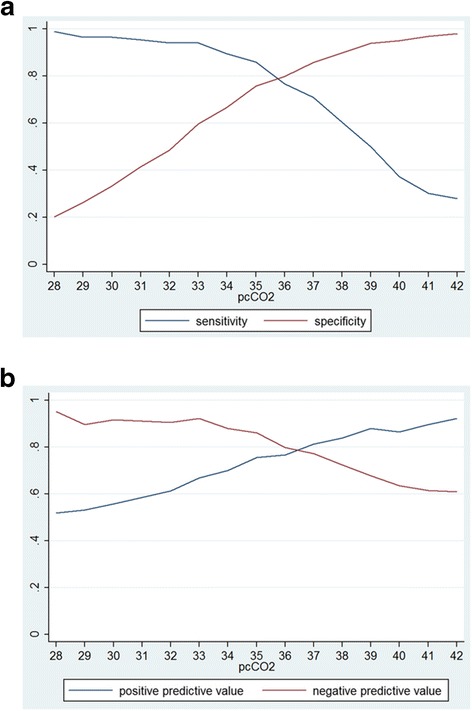
**Diagnostic performance of pCO**_**2**_ **. a)** Sensitivity and specificity of p_c_CO_2_ for a diagnosis of PH-HFpEF. **b)** Positive and negative predictive value of p_c_CO_2_ for a diagnosis of PH-HFpEF.

P_c_CO_2_ at time of diagnosis was 33 ± 4 mmHg in the IPAH group and 40 ± 5 mmHg in the PH-HFpEF group (p < 0.001), respectively (Figure 1). According to ROC analysis, a p_c_CO_2_ of 36 mmHg was the best discriminator between both entities with an area under curve of 0.868 (95% confidence interval, 0.816 – 0.920; p < 0.001; Figure 2). The lower p_c_CO_2_, the higher was the likelihood of IPAH and vice versa (Figure 3). P_c_CO_2_ values between 34 and 41 mmHg had limited discriminatory power, but P_c_CO_2_ values outside these margins provide valuable information. Assuming equal pre-test probability for each diagnosis, any P_c_CO_2_ < 34 mmHg excluded the presence of PH-HFpEF with a likelihood of >90%, whereas the likelihood of IPAH was <10% in patients with any P_c_CO_2_ > 41 mmHg (Figure 3). P_c_CO_2_ values >41 mmHg were found in 14% and P_c_CO_2_ levels <34 mmHg in 35% of the patients in this study, respectively; thus P_c_CO_2_ measurements provided relevant diagnostic information in 49% of the patients in this series.

### Correlations between p_c_CO_2_ and clinical variables

In patients in with IPAH, p_c_CO_2_ correlated with BMI and inversely with PVR. In the PH-HFpEF group, p_c_CO_2_ correlated with age at diagnosis and cardiac output. Even if statistically significant, all these correlations were weak (Table 2).

**Table 2:** **Correlations between p**_**c**_ **CO**_**2**_ **and hemodynamic variables**

	**IPAH**		**PH-HFpEF**	
	**r**	**p**	**r**	**p**
**Age at diagnosis**	0.062	0.544	0.432*	**<0.001**
**BMI**	**0.231**	**0.021**	0.057	0.630
**6MWD**	0.141	0.641	−0.022	0.846
**RA**	0.063	0.586	−0.012	0.923
**PAPm**	−0.144	0.155	−0.027	0.810
**PAWP**	0.051	0.638	0.033	0.766
**CO**	0.182	0.147	**0.296**	**0.028**
**CI**	0.052	0.658	**0.232**	**0.080**
**PVR**	**−0.278**	**0.009**	−0.203	0.105
**SvO**_**2**_	0.118	0.320	0.179	0.210
**PaO**_**2**_	0.087	0.612	0.123	0.534

*Bold numbers reflect statistically significant associations.

## Discussion

The present data confirm clinical observations that p_c_CO_2_ values tend to be lower in patients with IPAH compared to patients with PH-HFpEF. The average p_c_CO_2_ in patients with PH-HFpEF was 40 mmHg, i.e. in the normal range. In contrast, the average p_c_CO_2_ in patients with IPAH was 33 mmHg, i.e. markedly reduced compared to normal values. A p_c_CO_2_ of 36 mmHg was the best cut-off for discriminating between IPAH and PH-HFpEF. According to ROC analysis, the AUC was 0.868 for this value, suggesting that p_c_CO_2_ may be helpful in distinguishing between both conditions. The lower the p_c_CO_2_, the lower the likelihood of PH-HFpEF and vice versa.

The physiological explanation for the low p_c_CO_2_ in IPAH is not entirely clear. A previous study on patients with IPAH also found a low p_c_CO_2_ at the time of diagnosis [16]. The median p_c_CO_2_ in that study was 32 mmHg, i.e. very similar to the average value of 33 mmHg in the present IPAH population. In the previous study, there was a significant, albeit weak, correlation between p_c_CO_2_ and cardiac output [16], which was not found in the present IPAH population.

The patients with PH-HFpEF enrolled in the present series had rather severe PH with a PAPm of 47 ± 10 mmHg. The average transpulmonary gradient was 25 ± 10 mmHg, the diastolic gradient 10 ± 8 mmHg, and the PVR 471 ± 218 dyn · s · cm^−5^, indicating that the majority of these patients had a combined pre- and post-capillary form of PH [2,24]. This is a population of patients that may be easily misclassified as IPAH, and it may be particularly such patients in whom p_c_CO_2_ measurements may provide valuable information.

Our study has several strength and limitations. Strengths include the relatively large sample size of well-characterized patients, all of whom had undergone a rigorous diagnostic assessment including right heart catheterization at the time of diagnosis. Limitations include the single center design, the lack of a validation cohort and the fact that our study did not further elucidate the mechanisms causing hypocarbia in patients with IPAH. In addition, our HFpEF population was unique in that most of these patients suffered from severe PH, presumably owing to a referral bias as these patients were referred to our center for evaluation of PH, and not of HFpEF.

The fact that we recorded all pressure readings at end-expiration is in line with current recommendations [23,25]. Several experts have pointed out that this approach may result in an overestimation of these pressures, most importantly the PAWP [26,27]. However, we excluded patients with lung disease so that these differences should have been marginal in our patients. The fact that the mean PAWP in our HFpEF population was 21 mmHg compared to 8 mmHg in our IPAH population, is reassuring. Finally, our results may not be applicable to patients with additional confounders, which may affect p_c_CO_2_, such as morbid obesity or underlying lung disease.

## Conclusion

Our data show that p_c_CO_2_ is significantly lower in patients with IPAH compared to patients with PH-HFpEF and may help distinguishing between both conditions. Further studies are needed to determine the value of p_c_CO_2_ in the diagnostic work-up of patients with PH.

## Abbreviations

DZL: German Center of Lung Research
IPAH: Idiopathic pulmonary arterial hypertension
PH-HFpEF: Pulmonary hypertension due to heart failure with preserved ejection fraction
p_c_CO_2_: Capillary pCO_2_
ROC: Receiver operated characteristics
PAPm: Mean pulmonary arterial pressure
PAWP: Pulmonary arterial wedge pressure
PH: Pulmonary hypertension
PAH: Pulmonary arterial hypertension
CTEPH: Chronic thromboembolic pulmonary hypertension
LVEDP: Left ventricular enddiastolic pressure
SvO_2_: Mixed venous oxygen saturation
PVR: Pulmonary vascular resistance
CI: Cardiac index
TPG: Transpulmonary gradient
DPG: Diastolic pulmonary gradient
AUC: Area under the curve
paO_2_: Arterial oxygen tension
